# Cryptotanshinone differentially induces cell death in *ATP6V0D1*-deficient pancreatic cancer cells

**DOI:** 10.20517/cdr.2025.103

**Published:** 2025-08-27

**Authors:** Fangquan Chen, Junhao Lin, Xiutao Cai, Hu Tang, Shengfeng Li, Ruirui Liang, Rui Kang, Zhenhui Zhang, Daolin Tang, Jiao Liu

**Affiliations:** ^1^DAMP Laboratory, The Third Affiliated Hospital, Guangzhou Medical University, Guangzhou 510150, Guangdong, China.; ^2^Department of intensive care unit, The Second Affiliated Hospital of Guangzhou Medical University, Guangzhou 510260, Guangdong, China.; ^3^Department of Surgery, University of Texas Southwestern Medical Center, Dallas, TX 75390, USA.; ^4^Department of Critical Care Medicine, DAMP Laboratory, State Key Laboratory of Respiratory Disease, the Third Affiliated Hospital, Guangzhou Medical University, Guangzhou 510150, Guangdong, China.; ^5^DAMP Laboratory, Department of Critical Care Medicine, the Third Affiliated Hospital, Guangzhou Medical University, Guangzhou 510150, Guangdong, China.

**Keywords:** ATP6V0D1, cryptotanshinone, cell death, tumor heterogeneity

## Abstract

**Aim:** Dysregulation of tumor-suppressive pathways can lead to constitutive activation of multiple oncogenic signaling cascades. Such overactivation makes cancer cells highly dependent on these pathways, creating potential therapeutic vulnerabilities. Based on our previous findings and current data, genetic knockout of ATPase H^+^ transporting V0 subunit D1 (ATP6V0D1) - a key mediator of alkaliptosis - induces hyperactivation of oncogenic pathways, including signal transducer and activator of transcription 3 (STAT3)-mediated lysosomal pH regulation and AKT serine/threonine kinase (AKT) signaling. It also alters cellular responses to cryptotanshinone therapy. This study aimed to investigate how *ATP6V0D1* deficiency reshapes oncogenic signaling networks and cellular heterogeneity in pancreatic ductal adenocarcinoma (PDAC), while evaluating therapeutic strategies that exploit alkaliptosis-related vulnerabilities.

**Methods:** *ATP6V0D1*-deficient SW1990 and MIAPaCa2 cells were generated via gene knockdown. Cell viability and death following various treatments were assessed using CCK-8 and propidium iodide assays. Transcriptomic analysis was conducted to identify feedback signaling pathways, while Western blotting was used to measure expression of signaling proteins. Macropinocytosis was evaluated by TRITC-dextran uptake. Additionally, The Cancer Dependency Map (DepMap) database was analyzed to explore background differences between SW1990 and MIAPaCa2 cells.

**Results:** *ATP6V0D1* deletion led to overactivation of STAT3-mediated lysosomal pH regulation and AKT signaling; inhibition of these pathways restored alkaliptosis. Notably, cryptotanshinone selectively induced cell death in *ATP6V0D1*-deficient MIAPaCa2 cells but not SW1990 cells. Resistance in SW1990 cells was mediated by FGFR2 upregulation, which was reversed upon FGFR2 inhibition.

**Conclusion:** *ATP6V0D1* deficiency drives PDAC progression via dual mechanisms: compensatory oncogenic signaling (STAT3/AKT) and FGFR2-mediated cellular heterogeneity. While targeting these pathways may offer therapeutic potential, tumor heterogeneity remains a major clinical challenge.

## INTRODUCTION

Cancer arises from long-term exposure to adverse internal and external factors that cause genetic mutations or dysregulation, ultimately leading to malignant development^[1]^. One of the major challenges in cancer treatment is drug resistance, which often results from feedback activation of multiple signaling pathways that promote tumor progression and therapeutic resistance^[2,3]^. Frequently implicated feedback pathways include fibroblast growth factor receptor (FGFR)^[4]^, phosphatidylinositol-4,5-bisphosphate 3-kinase (PI3K)-AKT serine/threonine kinase (AKT)^[5]^, mitogen-activated protein kinase (MAPK)^[6]^, and signal transducer and activator of transcription 3 (STAT3), among others^[2]^. Dysregulation of oncogenes is typically responsible for the abnormal activation of these pro-survival signaling networks. Accordingly, targeting critical signaling nodes has emerged as a key strategy to overcome drug resistance^[3,5]^. For example, poly(ADP-ribose) polymerase (PARP) inhibitors exert synthetic lethal effects in cancer cells with inactivated BRCA1/2 DNA repair genes (e.g., ovarian, breast, and pancreatic cancers), and have shown significant clinical benefit. Thus, therapeutic strategies exploiting synthetic lethality induced by oncogene inactivation represent an important avenue in cancer research^[7]^. Nevertheless, tumor evolution frequently involves molecular and genetic alterations that drive heterogeneity in cell growth rates and drug sensitivities among cancer subtypes.

Alkaliptosis, a pH-dependent form of regulated cell death (RCD) triggered by the small molecule JTC801, is characterized by intracellular alkalinization-mediated cytotoxicity and lysosomal destabilization^[8-10]^. A key mechanism involves binding of the tumor suppressor ATPase H^+^ transporting V0 subunit D1 (ATP6V0D1) to STAT3, which disrupts STAT3-mediated lysosomal function and pH homeostasis. Genetic depletion of ATP6V0D1 abolishes this interaction, alleviating STAT3 inhibition and thereby conferring resistance to alkaliptosis^[8]^. Interestingly, treatment of ATP6V0D1-deficient pancreatic cancer cell lines (SW1990 and MIAPaCa2) with the selective STAT3 phosphorylation inhibitor cryptotanshinone showed distinct responses. Cryptotanshinone monotherapy at subtoxic concentrations (5 μM) induced selective cytotoxicity in *ATP6V0D1*-deficient MIAPaCa2 cells but had minimal effect on SW1990 cells under the same conditions^[8]^. However, combining cryptotanshinone with JTC801 restored alkaliptosis sensitivity in *ATP6V0D1*-knockdown cells. These subtype-specific outcomes highlight an unresolved mechanistic divergence in STAT3 pathway regulation that warrants further investigation.

In this study, we demonstrate that inhibition of the *ATP6V0D1* knockdown-dependent feedback pathway effectively restores cellular sensitivity to alkaliptosis. Notably, *ATP6V0D1* deletion exerted distinct effects in pancreatic ductal adenocarcinoma (PDAC) models, differentiating FGFR2-dependent (SW1990) from FGFR2-independent (MIAPaCa2) subtypes. Specifically, cryptotanshinone induced oxidative stress-mediated cell death in *ATP6V0D1*-deficient MIAPaCa2 cells, whereas inhibition of the FGFR2 pathway synergistically enhanced cryptotanshinone sensitivity in *ATP6V0D1*-knockdown SW1990 cells. Together, these findings reveal a previously unrecognized, tumor-heterogeneous resistance mechanism involving FGFR2 activation in *ATP6V0D1*-deficient PDAC cells.

## METHODS

### Reagents

The GAPDH antibody (AF7021; 1:5,000) was obtained from Affinity Biosciences. Antibodies against ATP6V0D1 (18274-1-AP; 1:1,000), Vinculin (26520-1-AP; 1:5,000), PARP1 (13371-1-AP; 1:1,000), and NQO1 (11451-1-AP; 1:1,000) were purchased from Proteintech Biotechnology. STAT3 (12640; 1:1,000), AKT (9272; 1:1,000), p-AKT (9271; 1:1,000), and p-STAT3 (9145; 1:1,000) antibodies were obtained from Cell Signaling Technology. Antibodies to FGFR2 (ab289968; 1:1,000), GSK3B (ab131356; 1:1,000), GPX4 (ab252833; 1:1,000), and C-MYC (ab32072; 1:1,000) were purchased from Abcam.

Chemical compounds including MK-2206 (S1078), LY2090314 (S7063), LY2874455 (S7057), Bovine serum albumin (E8149), Bafilomycin A1 (S1413), Ferrostatin-1 (S7243), Z-VAD-FMK (S7023), necrostatin-1 (S8073) N-acetylcysteine (S1623), Cryptotanshinone (S2285), Vitamin E (S4686), and JTC801 (S2722) were obtained from Selleck Chemicals. TRITC-dextran (42874) was obtained from Sigma Aldrich, and EIPA (HY-101840) was purchased from MedChemExpress. Chemical compounds were dissolved according to the manufacturer’s instructions, and final working concentrations were determined based on experimental requirements.

### Cell culture and drug preparation

MIAPaCa2 (CRL-1420), AsPC1 (CRL-1682), SW1990 (CRL-2172), and PANC1 (CRL-1469) cell lines were obtained from the American Type Culture Collection (ATCC). Cells were cultured in Dulbecco’s Modified Eagle Medium (DMEM) containing 10% fetal bovine serum (FBS; Thermo Fisher Scientific, A3840001) and 1% penicillin-streptomycin (Yeasen, 60162ES76), and maintained at 37 °C in a humidified incubator with 5% CO_2_. Cells were passaged when they reached 90% confluency. Mycoplasma contamination was checked every 3 months.

Experimental drugs were prepared according to the manufacturers’ instructions, with final concentrations kept below 0.01%.

### Western blot

Cells were collected and lysed in RIPA buffer (Cell Signaling Technology, 9803) containing a phosphatase inhibitor (Cell Signaling Technology, 5872) on ice for 30 min with occasional mixing. Lysates were centrifuged at 15,000 rpm for 15 min at 4 °C, and the supernatant was collected for protein quantification using the bicinchoninic acid (BCA) assay (Thermo Fisher Scientific, 23225). Proteins were denatured by boiling at 95 °C for 10 min.

Equal amounts of protein (30 µg) were separated by electrophoresis and transferred onto polyvinylidene fluoride membranes (Millipore, IPVH00010). Membranes were blocked with 5% skimmed milk at room temperature for 1 h and incubated with primary antibodies and placed on a shaker at 4 °C overnight. After washing 3-5 times with TBST (5 min each), membranes were incubated with horseradish peroxidase-conjugated secondary antibodies at room temperature for 1 h, followed by 5 washes with TBST. Signals were captured using a ChemiDoc Touch Imaging System (Bio-Rad) and analyzed with Image Lab software (Bio-Rad).

### RNA interference (RNAi)

Human *ATP6V0D1*-shRNA1 (5′-CCAGCTTCCTAGACTTCATTA-3′), *ATP6V0D1*-shRNA2 (5′-GCACGAGGTAAAGCTGAACAA-3′), and control empty shRNA (pLKO.1) were obtained from Quanyang Biotechnology. High-purity adenovirus particles were generated using 293T cells. Briefly, 90% confluent 293T cells were cultured in Opti-MEM (Gibco, 31985070) without FBS for 1 h. Plasmids [shRNA (1,600 ng), pSPAX2 (1,200 ng), and pMD2G (400 ng)] were transfected, and after 8 h, the medium was replaced with DMEM containing 10% FBS and cultured overnight. Cells were then cultured in DMEM with 20% FBS for 24 h. Viral supernatants were collected, centrifuged at 1,500 × *g* for 5 min, and added to target cells for 48 h. Transfected cells were selected with graded concentrations of puromycin (2 μg/mL; YEASEN, 60210ES72) for 10 days.

For control siRNA experiments, GSK3B siRNAs were obtained from GenePharma. FGFR2-specific siRNAs, 5′-GGGAGUACUUGCAGAUAAAGG-3′ (siRNA-1) and 5′-GUAGGACUGUAGACAGUGAAA-3′ (siRNA-2), were obtained from Sigma-Aldrich. siRNAs were transfected into PDAC cells using Lipofectamine RNAiMAX (Thermo Fisher Scientific, 13778500) according to the manufacturer’s instructions, and functional assays and protein validation were performed 72 h post-transfection. The GSK3B siRNA sense was 5′-GCUAGAUCACUGUAACAUAGU-3′.

### Cell viability assay

Cells in the logarithmic growth phase were seeded in 96-well plates at approximately 5,000 cells per well. After 24 h of growth, drugs were added according to the experimental design and incubated for the required duration. Subsequently, the medium was removed, and a 10% CCK-8 solution in serum-free DMEM was added. Plates were incubated in a cell culture incubator for 0.5-4 h, protected from light. Absorbance was measured at 450 nm using a multifunctional microplate reader. Optical density at 450 nm (OD450) was recorded, showing a linear correlation with the viable cell count. Cell survival rates were calculated as percentages normalized to control wells, with full viability (100%) defined as an OD450 of 1.

### Cell death assay

Cells in the logarithmic growth phase were seeded in 6-well plates at approximately 3 × 10^6^ cells per well. After 24 h, cells were treated with drugs according to the experimental protocol. Propidium iodide (PI) and Hoechst 33342 (Bestbio; BB-4131) were added to the culture wells at a 1:1,000 dilution and incubated in the dark for 30-40 min. Cells were observed using a fluorescence microscope. Quantification was performed using ImageJ software, and the ratio of PI-positive cells to Hoechst 33342-positive cells was calculated for each well.

### TRITC-dextran uptake assay

Following previously described methods^[11]^, cells were seeded in confocal dishes and treated according to the experimental protocol. Macropinosomes were visualized using high molecular weight TRITC-dextran (42874; Sigma Aldrich). TRITC-dextran was added to serum-free medium at a final concentration of 1 mg/mL and incubated with cells at 37 °C for 30 min. Cells were washed five times with cold PBS (phosphate-buffered saline) and rapidly fixed in 4% formaldehyde (119690010, Thermo Fisher Scientific). Imaging was performed using a fluorescence confocal microscope. Image analysis was conducted in ImageJ using the “Analyze Particles” function. Cup-shaped structures were manually selected with the polygon tool, and threshold segmentation (Process > Binary > Watershed) was applied to separate adjacent particles. Data were obtained from at least three randomly selected fields per sample to quantify cumulative particle area.

### Transcriptomics

Cultured cells (1 × 10^6^) were homogenized in TRIzol reagent (Invitrogen) for total RNA extraction. RNA integrity was confirmed using an Agilent 2100 Bioanalyzer [RNA integrity number (RIN) > 8.0]. Polyadenylated mRNA was enriched via poly(A) selection using the NEBNext Poly(A) mRNA Magnetic Isolation Module (New England Biolabs). Strand-specific cDNA libraries were prepared using the KAPA Stranded mRNA-Seq Kit (Roche) following the manufacturer’s protocols. Fragmented mRNA (300-500 bp) underwent first-strand synthesis with random hexamers and Actinomycin D to suppress second-strand synthesis. Double-stranded cDNA was subjected to end repair, A-tailing, and adapter ligation. Libraries were amplified with 12 PCR cycles and quantified with a Qubit 4.0 Fluorometer (Thermo Fisher Scientific). Paired-end sequencing (150 bp) was performed on an Illumina NovaSeq 6000 platform, generating ≥ 40 million reads per sample. Raw reads were quality-filtered using FastQC v0.11.9 and trimmed with Trimmomatic v0.39 (SLIDINGWINDOW:4:20, MINLEN:50). Processed reads were aligned to the GRCh38/hg38 reference genome using STAR v2.7.10a with two-pass mode for splice junction detection. Transcript quantification was performed using featureCounts v2.0.3, and differentially expressed genes (DEGs) were identified using DESeq2 v1.34.0 (FDR-adjusted *P* < 0.05, |log2 fold change| > 1). Functional enrichment was analyzed via Metascape using Gene Ontology (GO) and Kyoto Encyclopedia of Genes and Genomes (KEGG) databases.

### Bioinformatics analysis

The DepMap database (https://depmap.org/portal/) was used to evaluate the biological background and metastatic potential of PDAC cells. Differential gene data following CRISPR knockout were derived from (DepMap Public 24Q4 + Score, Chronos. MetMap500 was employed to analyze the metastatic potential of PDAC cells by assessing DNA barcode abundance in each organ relative to the initial population. Data were presented on a log_10_ scale ranging from -4 to 4: ≤ -4, non-metastatic; -4 to -2, weakly metastatic with low confidence; ≥ -2, metastatic with higher confidence.

### Measurement of lipid ROS

Lipid ROS levels were measured using the BODIPY 581/591 C11 probe (Thermo Fisher Scientific, D3861). Cells were incubated with 5 µM probe for 30 min at 37 °C (5% CO_2_). After incubation, cells were washed 3-4 times with PBS, trypsinized, and resuspended in PBS for analysis. Oxidation of the probe shifted fluorescence from ~590 to ~510 nm. Fluorescence was quantified using a BD Accuri C6 Plus flow cytometer (BD Biosciences), and data were analyzed using FlowJo 10. A minimum of 10,000 events per sample were collected.

### Statistical analysis

Data are presented as mean ± SEM. Analyses were performed using GraphPad Prism 8.02, and figures were prepared using Adobe Illustrator 2023. Comparisons between two groups were conducted using unpaired Student’s *t*-tests. For multiple group comparisons, one-way or two-way analysis of variance (ANOVA) followed by Tukey’s multiple comparison test was applied. All experiments were conducted independently at least three times. Statistical significance was defined as *P* < 0.05.

## RESULTS

### Macropinocytosis is not responsible for cryptotanshinone-induced differential sensitization of PDAC cells

**Figure 1:**
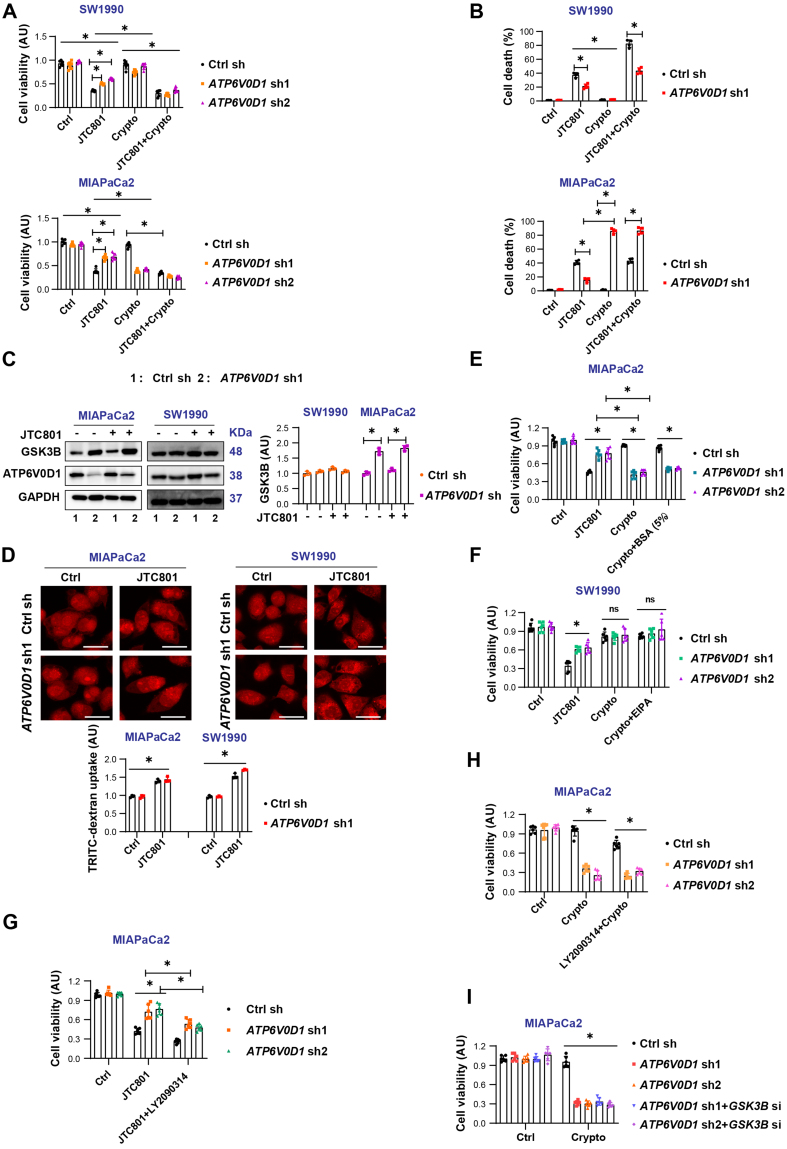
Macropinocytosis is not responsible for cryptotanshinone-induced differential sensitization of PDAC cells. (A) CCK-8 assay assessing cell viability after treatment with JTC801, cryptotanshinone (Crypto), or their combination (Drug concentrations: JTC801, 3.5 μM; Crypto, 5 μM; treatment time: 24 h); (B) Cell death assay evaluating the proportion of dead cells under the indicated treatments (Same concentrations and treatment time as A); (C) Western blot analysis of protein expression in the indicated groups (Drug concentration: JTC801, 3.5 μM; treatment time: 24 h); (D) TRITC-dextran uptake to assess macropinocytosis in the indicated cell lines (Drug concentration: JTC801, 3.5 μM; treatment time: 24 h). Scale bar = 50 µm; (E) CCK-8 assay assessing cell viability after treatment with JTC801, Crypto, or Crypto combined with 5% BSA; (F) CCK-8 assay evaluating cell viability after treatment with JTC801, Crypto, or Crypto combined with EIPA (Drug concentrations: JTC801, 3.5 μM; Crypto, 5 μM; EIPA, 5 μM; treatment time: 24 h); (G and H) CCK-8 assay assessing the effect of LY2090314 combined with JTC801 (G) or Crypto (H) on cell viability (Drug concentrations: JTC801, 3.5 μM; Crypto, 5 μM; LY2090314, 5 μM; treatment time: 24 h); (I) CCK-8 assay analyzing MIAPaCa2 cell viability treated with Crypto (5 μM) with *ATP6V0D1* knockout alone or simultaneous *GSK3B* knockout. Data (A and B, D-I) are presented as mean ± SD from ≥ 3 biologically independent samples (A, *n* = 6; B = 4; D, *n* = 3; E-I, *n* = 6). Statistical significance was determined using two-way ANOVA with Tukey’s multiple comparisons test. Western blot data (C) represent three independent experiments. Source data are provided. ^*^P < 0.05; ns: not significant. PDAC: Pancreatic ductal adenocarcinoma; ATP6V0D1: ATPase H^+^ transporting V0 subunit D1; SD: standard deviation; ANOVA: analysis of variance.

Our previous studies showed that JTC801 induced alkaliptosis by targeting ATP6V0D1 in PDAC cells [Supplementary Figure 1A]. Knockdown of *ATP6V0D1* alleviated JTC801-induced alkaliptosis via STAT3-mediated regulation of lysosomal pH^[8]^. Consequently, inhibition of STAT3 restored JTC801 sensitivity in *ATP6V0D1* knockdown PDAC cells, either pharmacologically (with cryptotanshinone) or genetically. Interestingly, treatment with cryptotanshinone alone significantly induced cell death in *ATP6V0D1*-deficient MIAPaCa2 cells but not in SW1990 cells [Figure 1A and B, Supplementary Figure 1B]. Analysis of the CRISPR whole-gene knockout dataset from the DepMap database revealed that the top 10 genes essential for proliferation and survival differ between MIAPaCa2 and SW1990 cells [Supplementary Figure 1C]. Additionally, the radial migration potential of the two cell lines was distinct [Supplementary Figure 1D], suggesting that tumor heterogeneity affects drug sensitivity and potentially clinical prognosis.

Considering that MIAPaCa2 cells represent a nutrient-deficient pancreatic tumor type^[12]^, extracellular proteins serve as an important nutrient source, either through uptake of monomeric amino acids or via macropinocytosis followed by lysosomal catabolism^[13,14]^. Glycogen synthase kinase 3 beta (GSK-3β) is known to inhibit micropinocytosis^[15]^. We found that GSK3B was significantly upregulated in *ATP6V0D1*-knockdown MIAPaCa2 cells, but not in SW1990 cells [Figure 1C]. To evaluate the effect of GSK-3β expression on macropinocytosis, we used tetramethylrhodamine isothiocyanate-labeled dextran (TRITC-dextran), a well-established marker of micropinocytosis^[13]^. No significant differences in macropinocytosis were observed between MIAPaCa2 and SW1990 cells [Figure 1D]. Supplementation with 5% FBS did not alleviate cryptotanshinone-induced toxicity in *ATP6V0D1*-knockdown MIAPaCa2 cells [Figure 1E]. Likewise, co-treatment with the macropinocytosis inhibitor EIPA did not significantly increase the sensitivity of *ATP6V0D1*-knockdown SW1990 cells to cryptotanshinone [Figure 1F]. In contrast, inhibition of GSK3B, either pharmacologically (LY2090314) or genetically, partially restored JTC801 sensitivity in *ATP6V0D1*-knockdown MIAPaCa2 cells [Figure 1G and H, Supplementary Figure 1E-G], but had no effect on cryptotanshinone-mediated cell death in these cells [Figure 1I].

Together, these results suggest that macropinocytosis does not account for the differential sensitivity of *ATP6V0D1*-deficient PDAC cells to cryptotanshinone. The observed differences in GSK3B expression likely represent an adaptive response to tumor heterogeneity rather than a determinant of drug sensitivity.

### The PI3K-AKT signaling induces alkaliptosis resistance but does not affect cryptotanshinone sensitivity

**Figure 2:**
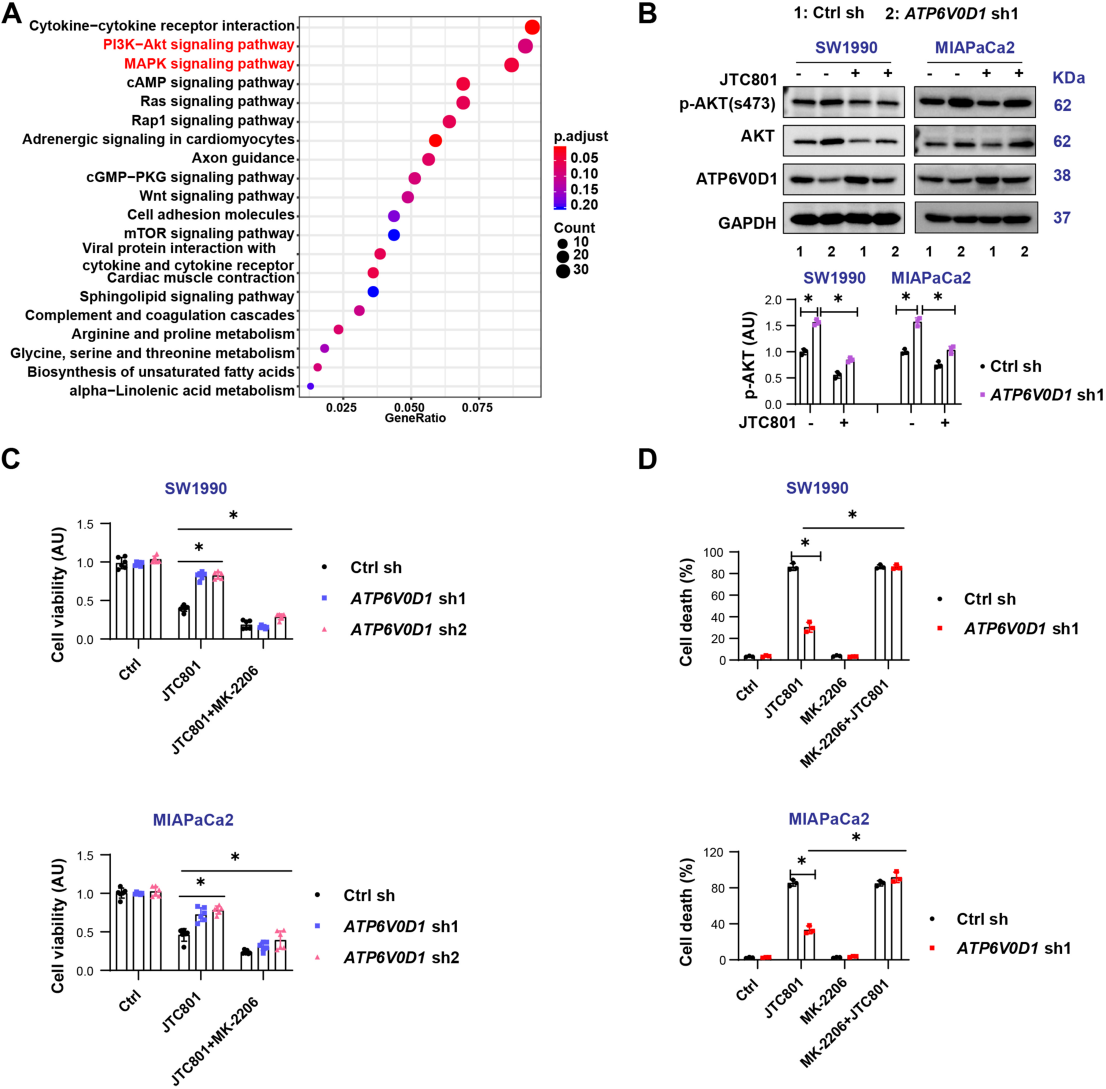
PI3K-AKT signaling induces alkaliptosis resistance but does not affect cryptotanshinone sensitivity. (A) KEGG pathway analysis of DEGs based on mRNA transcriptomics; (B) Western blot analysis of indicated proteins in the indicated groups (Drug concentration: JTC801, 3.5 μM; treatment time: 24 h); (C) CCK-8 assay measuring cell viability after treatment with JTC801 or Crypto combined with MK-2206 (Drug concentrations: JTC801, 3.5 μM; MK-2206, 2 μM; treatment time: 24 h); (D) Cell death assay showing the proportion of dead cells following treatment with JTC801, MK-2206, or their combination (same concentrations and time as above). Data (C and D) are shown as mean ± SD from three or more biologically independent samples (C, *n* = 6; D, *n* = 3). Statistical significance was determined using two-way ANOVA with Tukey’s multiple comparisons test. Western blots (B) represent three independent experiments. Source data are provided. ^*^P < 0.05 was considered statistically significant; ns: not significant. PI3K: Phosphatidylinositol-4,5-bisphosphate 3-kinase; AKT: AKT serine/threonine kinase; KEGG: Kyoto Encyclopedia of Genes and Genomes; DEGs: differentially expressed genes; SD: standard deviation; ANOVA: analysis of variance.

To further investigate the mechanisms underlying this variability, we performed mRNA transcriptomic analysis in MIAPaCa2 cells [Supplementary Figure 2A]. The results revealed significant alterations in the PI3K/AKT and MAPK pathways [Figure 2A]. Western blot analysis showed that knockdown of *ATP6V0D*1 markedly increased AKT phosphorylation in both SW1990 and MIAPaCa2 cells [Figure 2B]. Notably, the two cell lines exhibited different AKT responses to JTC801, which may reflect differences in their genetic backgrounds and inherent sensitivity to JTC801. Since PI3K-AKT signaling is critical for cancer cell proliferation, survival, and signal transduction, inhibition of AKT phosphorylation with the inhibitor MK-2206 restored JTC801 sensitivity [Figure 2B-D, Supplementary Figure 2B and C], but did not significantly affect cryptotanshinone sensitivity in SW1990 cells [Supplementary Figure 2D].

Together, these findings indicate that PI3K-AKT signaling mediates resistance to alkaliptosis but is not responsible for differential cryptotanshinone sensitivity.

### Differential expression of FGFR2 partially determines cryptotanshinone sensitivity in *ATP6V0D1*-deficient cells

FGFR is frequently activated in gastrointestinal cancers, including pancreatic cancer, in both ligand-dependent and ligand-independent manners, leading to activation of downstream pro-survival pathways such as PI3K-AKT and MAPK^[16]^, consistent with our transcriptomic results [Figure 2A]. Among FGFR family members, FGFR2 plays a critical role in cancer progression through activating mutations, fusions, or amplifications^[16]^.

**Figure 3:**
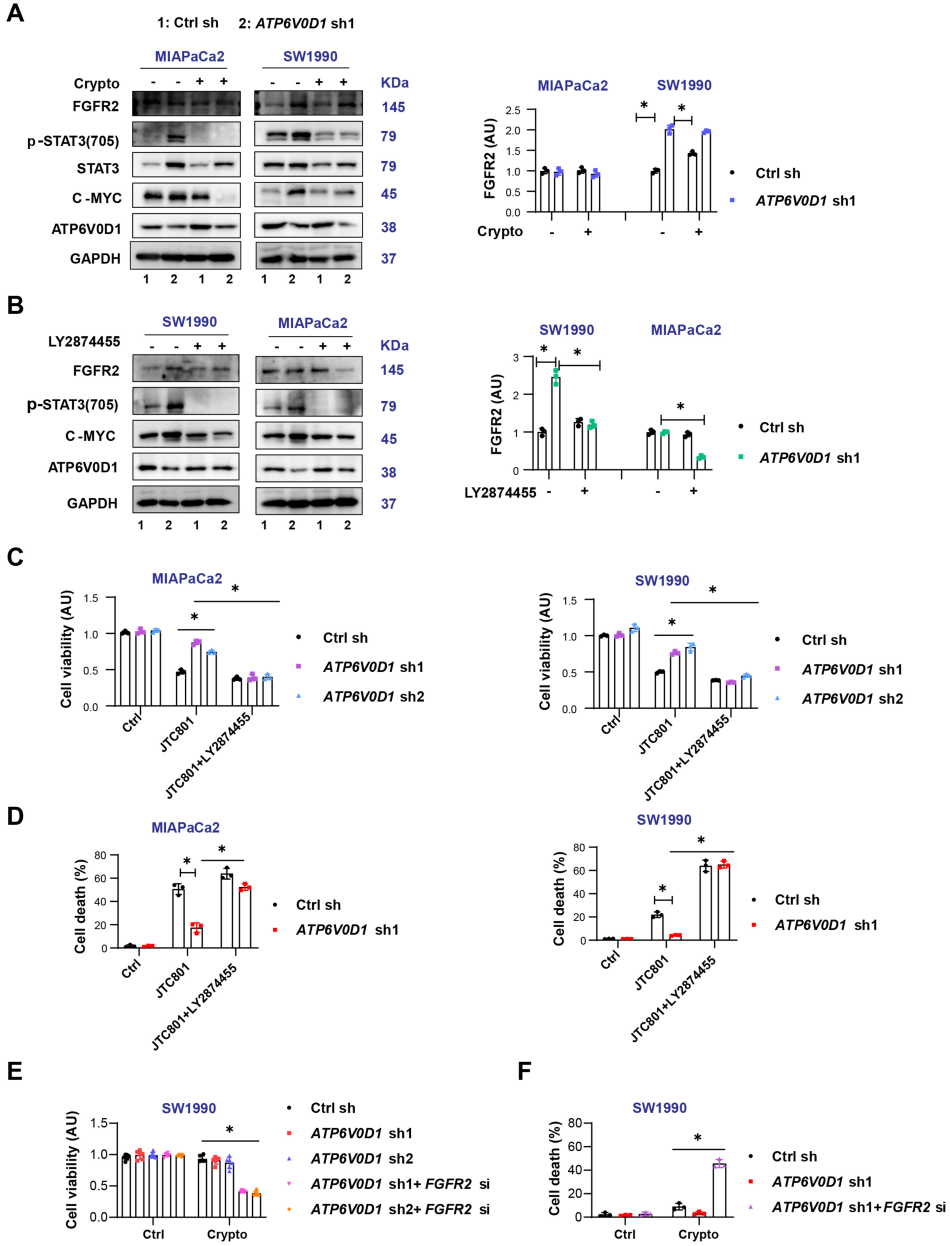
Differential FGFR2 expression partially determines cryptotanshinone sensitivity in *ATP6V0D1*-deficient cells. (A and B) Western blot analysis of indicated proteins after treatment (Drug concentrations: JTC801, 3.5 μM; Crypto, 5 μM; LY2874455, 450 nM; treatment time: 24 h); (C) CCK-8 assay of cell viability after JTC801, LY2874455, or combination treatment (same concentrations and time as above); (D) Cell death assay showing mortality after JTC801, LY2874455, or combination treatment; (E) CCK-8 assay evaluating cell viability of SW1990 cells treated with Crypto (5 μM) after *ATP6V0D1* knockout or combined *ATP6V0D1* and *FGFR*2 knockout; (F) Cell death assay for the indicated groups (Drug concentration: Crypto, 5 μM; treatment time: 24 h). Data (C-E) are shown as mean ± SD from three or more biologically independent samples (C and D, *n* = 3; E, *n* = 6; F, *n* = 3). Statistical significance was performed using two-way ANOVA with Tukey’s multiple comparisons test. Western blots (A and B) represent three independent experiments. Source data are provided. ^*^P < 0.05 was considered statistically significant; ns: not significant. FGFR2: Fibroblast growth factor receptor 2; ATP6V0D1: ATPase H^+^ transporting V0 subunit D1; SD: standard deviation; ANOVA: analysis of variance.

Western blot analysis showed that ATP6V0D1 knockdown upregulated FGFR2 in SW1990 cells but not in MIAPaCa2 cells [Figure 3A]. Total STAT3 protein levels also differed between the two cell lines, potentially due to cell type-specific effects, interactions between ATP6V0D1 and STAT3, or compensatory signaling pathways. Cryptotanshinone did not alter FGFR2 expression [Figure 3A]. Inhibition of FGFR2 with LY2874455 in *ATP6V0D1*-knockdown cells reduced STAT3 phosphorylation and c-MYC expression and restored JTC801-mediated inhibition of cell viability and induction of cell death [Figure 3B-D, Supplementary Figure 3A and B]. Importantly, genetic inhibition of FGFR2 in *ATP6V0D1*-knockdown SW1990 cells partially sensitized the cells to cryptotanshinone [Figure 3E and F, Supplementary Figure 3C]. Cryptotanshinone preferentially kills cells with high NAD(P)H quinone dehydrogenase 1 (NQO1) expression^[17]^; however, NQO1 levels were comparable between SW1990 and MIAPaCa2 cells after *ATP6V0D1* knockdown [Supplementary Figure 3D]. Additionally, *ATP6V0D1* knockdown induced phosphorylation of STAT3 at Tyr705 in AsPC1 cells, while cryptotanshinone alone partially induced cell death in these knockdown cells [Supplementary Figure 3E and F].

Collectively, these results suggest that FGFR2 activation contributes, at least in part, to the differential sensitivity of *ATP6V0D1*-knockdown cells to cryptotanshinone.

### Cryptotanshinone induces ROS-dependent cell death in *ATP6V0D1*-deficient MIAPaCa2 cells

**Figure 4:**
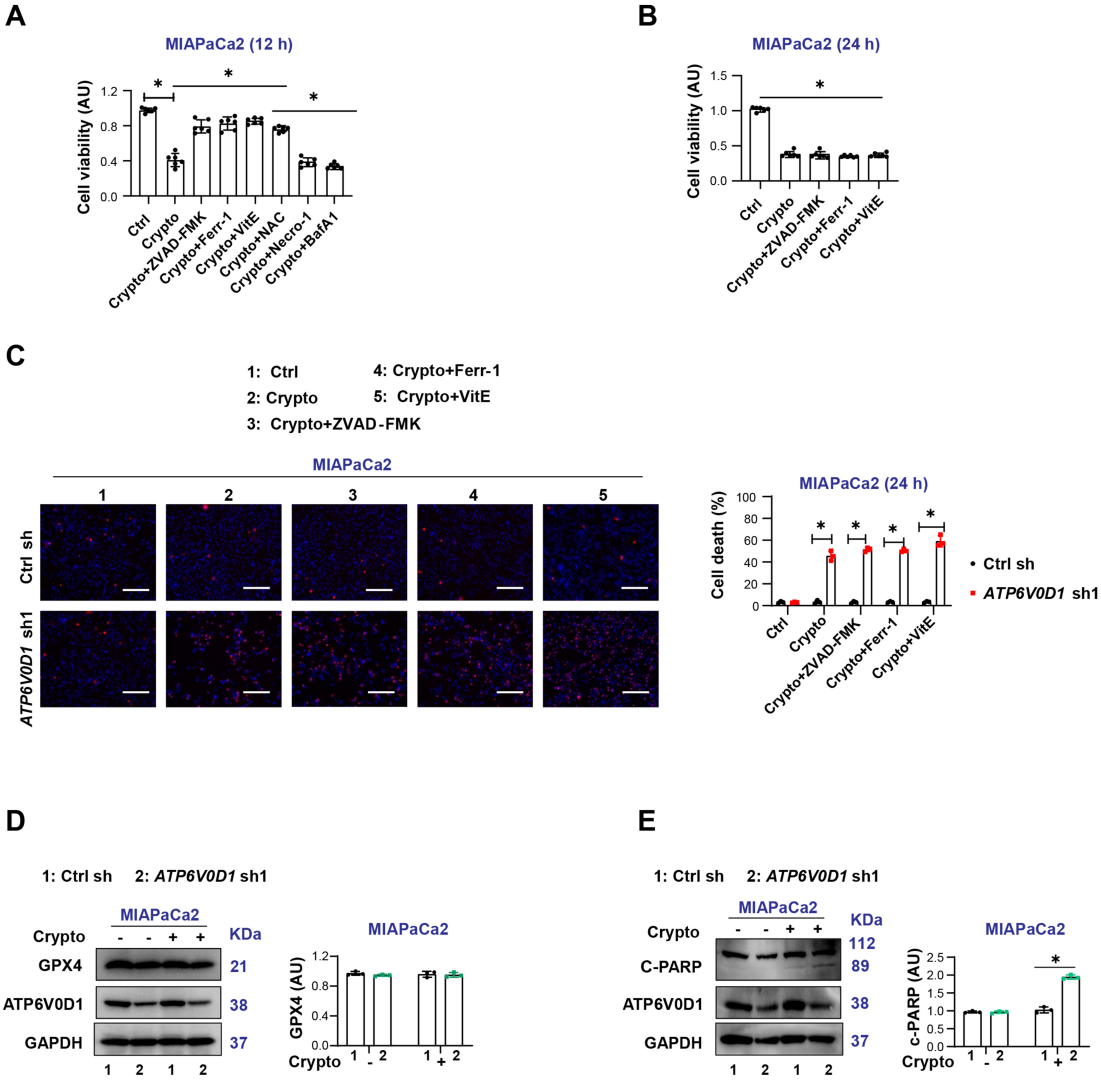
Differential expression of FGFR2 partially determines cryptotanshinone sensitivity in *ATP6V0D1*-deficient cells. (A) CCK-8 assay showing cell viability after treatment with Crypto alone or in combination with various cell death inhibitors (Drug concentrations: Crypto, 5 μM; ZVAD-FMK, 50 μM; Ferr-1, 500 nM; VitE, 5 mM; NAC, 5 mM; Necro-1, 500 nM; Baf A1, 500 nM; treatment time: 12 h); (B) CCK-8 assay showing cell viability after treatment with Crypto alone or in combination with inhibitors (Drug concentrations: Crypto, 5 μM; ZVAD-FMK, 50 μM; Ferr-1, 500 nM; VitE, 5 mM; treatment time: 24 h); (C) Cell death assay showing the proportion of dead cells following treatment with Crypto alone or in combination with inhibitors (Drug concentrations: Crypto, 5 μM; ZVAD-FMK, 50 μM; Ferr-1, 500 Nm, VitE, 5 mM; treatment time: 24 h). Scale bar = 200 µm; (D and E) Western blot analysis showing protein expression in the indicated groups (Drug concentration: Crypto, 5 μM; treatment time: 24 h). Data (A-C) are shown as mean ± SD from at least three biologically independent samples (A and B, *n* = 6; C, *n* = 3). Statistical significance was analyzed using two-way ANOVA with Tukey’s multiple comparisons test. Western blots (D and E) represent three independent experiments. Source data are provided. ^*^P < 0.05 was considered statistically significant; ns: not significant. FGFR2: Fibroblast growth factor receptor 2; ATP6V0D1: ATPase H^+^ transporting V0 subunit D1; SD: standard deviation; ANOVA: analysis of variance.

To further determine the pathway through which cryptotanshinone induces cell death, we treated *ATP6V0D1*-knockdown MIAPaCa2 cells with cryptotanshinone in the absence or presence of inhibitors of various RCD pathways. CCK-8 assays showed that the inhibitors of apoptosis (ZVAD-FMK), ferroptosis (Ferr-1), and ROS scavengers [Vitamin E and N-acetyl cysteine (NAC)] rescued cryptotanshinone-induced reduction in cell viability at 12 h. In contrast, inhibitors of necroptosis (necrostatin-1, Necro-1) and autophagy (Bafilomycin A1, Baf A1) had no effect [Figure 4A]. Notably, the protective effects of these inhibitors were lost at 24 h [Figure 4B and C].

Accumulating evidence suggests that cryptotanshinone can promote ROS production and mitochondria-mediated apoptosis in cancer cells^[18,19]^. Consistently, Western blot analysis revealed that cryptotanshinone induced apoptosis-associated molecular events, including poly (ADP-ribose) polymerase (c-PARP) cleavage, but did not trigger ferroptosis, as indicated by the absence of glutathione peroxidase 4 (GPX4) degradation [Figure 4D and E]. Furthermore, treatment with cryptotanshinone for 12 h induced lipid ROS accumulation in ATP6V0D1-deficient MIAPaCa2 cells, which was alleviated by Ferr-1 [Supplementary Figure 3G].

Taken together, these results suggest that cryptotanshinone activates multiple cell death pathways in *ATP6V0D1*-deficient MIAPaCa2 cells.

## DISCUSSION

The loss of tumor suppressors can create cancer-specific vulnerabilities that may be exploited for therapeutic purposes^[20,21]^. A classic example is the selective efficacy of PARP inhibitors in tumors harboring BRCA1/2 mutations, which has significantly advanced therapeutic strategies for ovarian and breast cancers^[22,23]^. These findings have also facilitated the development of combination therapies leveraging similar mechanisms. However, unlike oncogenes, tumor suppressor deletions do not directly provide actionable targets for cancer therapy. The genetic and transcriptional profiles of cancer cell lines are highly heterogeneous, and even within a single tumor, malignant cell populations are not homogeneous. In this study, we found that inhibition of ATP6V0D1 activated multiple oncogenic pathways via feedback mechanisms and elicited distinct responses to cryptotanshinone in different PDAC cell lines.

Mutations or deletions in the targets of single-agent therapies often severely compromise antitumor efficacy, and the concept of synthetic lethality has provided a new perspective on cancer treatment. However, due to the genetic heterogeneity of cancer cells, it remains unclear which feedback mechanisms are critical for controlling treatment tolerance. Our current evidence indicates that *ATP6V0D1* knockdown induces alkaliptosis resistance in PDAC cells (MIAPaCa2 and SW1990). Mechanistically, this resistance involves STAT3-mediated lysosomal pH homeostasis and AKT activation. Consequently, dual inhibition of STAT3 and ATK restores sensitivity to alkaliptosis. Interestingly, pharmacological inhibition of STAT3 phosphorylation in *ATP6V0D1*-deficient MIAPaCa2 cells induced significant cell death even in the absence of JTC801, whereas it was ineffective in SW1990 cells. In SW1990 cells, *ATP6V0D1* knockdown led to specific activation of FGFR2, which mitigated the cytotoxic effects of cryptotanshinone, highlighting the influence of PDAC cell heterogeneity on drug sensitivity.

Notably, FGFR2 inhibition only partially restored cryptotanshinone sensitivity, suggesting possible involvement of other FGFRs (e.g., FGFR1, FGFR3, or FGFR4) or alternative feedback mechanisms. In addition, ATP6V0D1 knockdown activated the MAPK pathway. This may reflect MAPK’s established role in promoting cell survival or changes in lysosomal homeostasis. For example, in macrophages, MAPK signaling regulates the expression of lysosomal acidification genes (e.g., ATP6V1H, ATP6V0D2) to maintain lysosomal function^[24]^. Additionally, apoptosis inhibitors and antioxidants only partially attenuated the anticancer activity of cryptotanshinone and were ineffective at later stages, indicating the coexistence of multiple cell death pathways, potentially mediated by lysosomal damage. Lysosomal membrane permeabilization is known to contribute to various cell death processes through the release of lysosomal contents^[25,26]^. AKT inhibition restored alkaliptosis sensitivity in *ATP6V0D1*-knockdown cells, likely by disrupting AKT-mediated lysosomal pH homeostasis. Recent studies have shown that AKT phosphorylates and stabilizes mucolipin TRP cation channel 1 (MCOLN1/TRPML1), promoting lysosomal efflux and inhibiting ferroptosis^[27]^. This suggests that AKT inhibition may destabilize lysosomal pH-regulated proteins, although the precise mechanisms remain unclear. It is important to note that our study only compared SW1990 and MIAPaCa2 cells, and it remains to be determined whether other pancreatic cancer lines or non-PDAC cell lines exhibit similar heterogeneity in *ATP6V0D1*-associated drug sensitivity.

*In vivo* validation is essential for advancing translational applications. However, this study primarily relied on *in vitro* models to investigate the role of *ATP6V0D1* deficiency in PDAC. While our findings provide mechanistic insights into compensatory activation of STAT3/AKT pathways and FGFR2-mediated cellular heterogeneity, the lack of *in vivo* validation remains a key limitation. The tumor microenvironment *in vivo* is highly complex, encompassing interactions among cancer cells, immune cells, stromal components, and the extracellular matrix, which cannot be fully recapitulated by two-dimensional cell culture. Additionally, our experimental setup does not account for systemic factors such as drug metabolism, toxicity, or organism-level tumor heterogeneity.

The differential responses of SW1990 and MIAPaCa2 cells to cryptotanshinone and FGFR2 inhibition may reflect their distinct genetic or epigenetic backgrounds. Future studies should incorporate mouse xenograft or patient-derived xenograft (PDX) models to validate the *in vivo* relevance of these pathways and assess the therapeutic feasibility of targeting *ATP6V0D1*-associated mechanisms.

In summary, our results indicate that alkaliptosis resistance in ATP6V0D1-deficient PDAC cells is mediated by STAT3-driven lysosomal pH regulation and AKT signaling, while PDAC cell heterogeneity contributes to variable sensitivity to cryptotanshinone, at least *in vitro*. Expanding *in vivo* studies will be crucial for bridging the gap between preclinical findings and translational applications.

## DECLARATIONS

### Availability of data and materials

The raw data supporting the findings of this study are available within this Article and its Supplementary Materials. Further data are available from the corresponding authors upon reasonable request.

### Copyright

© The Author(s) 2025.
