# Supplementary material for: Cryptotanshinone differentially induces cell death in ATP6V0D1-deficient pancreatic cancer cells
Source: Cancer Drug Resist. 2025 Aug 27;8:45. doi: 10.20517/cdr.2025.103 (PMC12462395; doi:10.20517/cdr.2025.103)
Supplement: Supplementary file 1 [file cdr-8-45-SupplementaryMaterials.pdf]

Supplementary Materials

Cryptotanshinone differentially induces cell death in *ATP6V0D1*-deficient pancreatic cancer cells

Fangquan Chen[1], Junhao Lin[1], Xiutao Cai[1], Hu Tang[1], Shengfeng Li[2], Ruirui Liang[1], Rui Kang[3], Zhenhui Zhang[4], Daolin Tang[3], Jiao Liu[5]

[1]DAMP Laboratory, The Third Affiliated Hospital, Guangzhou Medical University, Guangzhou 510150, Guangdong, China.
[2]Department of intensive care unit, The Second Affiliated Hospital of Guangzhou Medical University, Guangzhou 510260, Guangdong, China.
[3]Department of Surgery, University of Texas Southwestern Medical Center, Dallas, TX 75390, USA.
[4]Department of Critical Care Medicine, DAMP Laboratory, State Key Laboratory of Respiratory Disease, the Third Affiliated Hospital, Guangzhou Medical University, Guangzhou 510150, Guangdong, China.
[5]DAMP Laboratory, Department of Critical Care Medicine, the Third Affiliated Hospital, Guangzhou Medical University, Guangzhou 510150, Guangdong, China.

Correspondence to: Prof. Daolin Tang, Department of Surgery, University of Texas Southwestern Medical Center, 5323 Harry Hines Blvd, Dallas, TX 75390. USA. E-mail: daolin.tang@utsouthwestern.edu; Prof. Jiao Liu, DAMP Laboratory, Department of Critical Care Medicine, the Third Affiliated Hospital, Guangzhou Medical University, No. 63 Duobao Road, Liwan District, Guangzhou 510150, Guangdong, China. E-mail: 2018683073@gzhmu.edu.cn; Prof. Zhenhui Zhang, Department of Critical Care Medicine, DAMP Laboratory, State Key Laboratory of Respiratory Disease, the Third Affiliated Hospital, Guangzhou Medical University, No. 63 Duobao Road, Liwan District, Guangzhou 510150, Guangdong, China. E-mail: zhangzhenhui@gzhmu.edu.cn

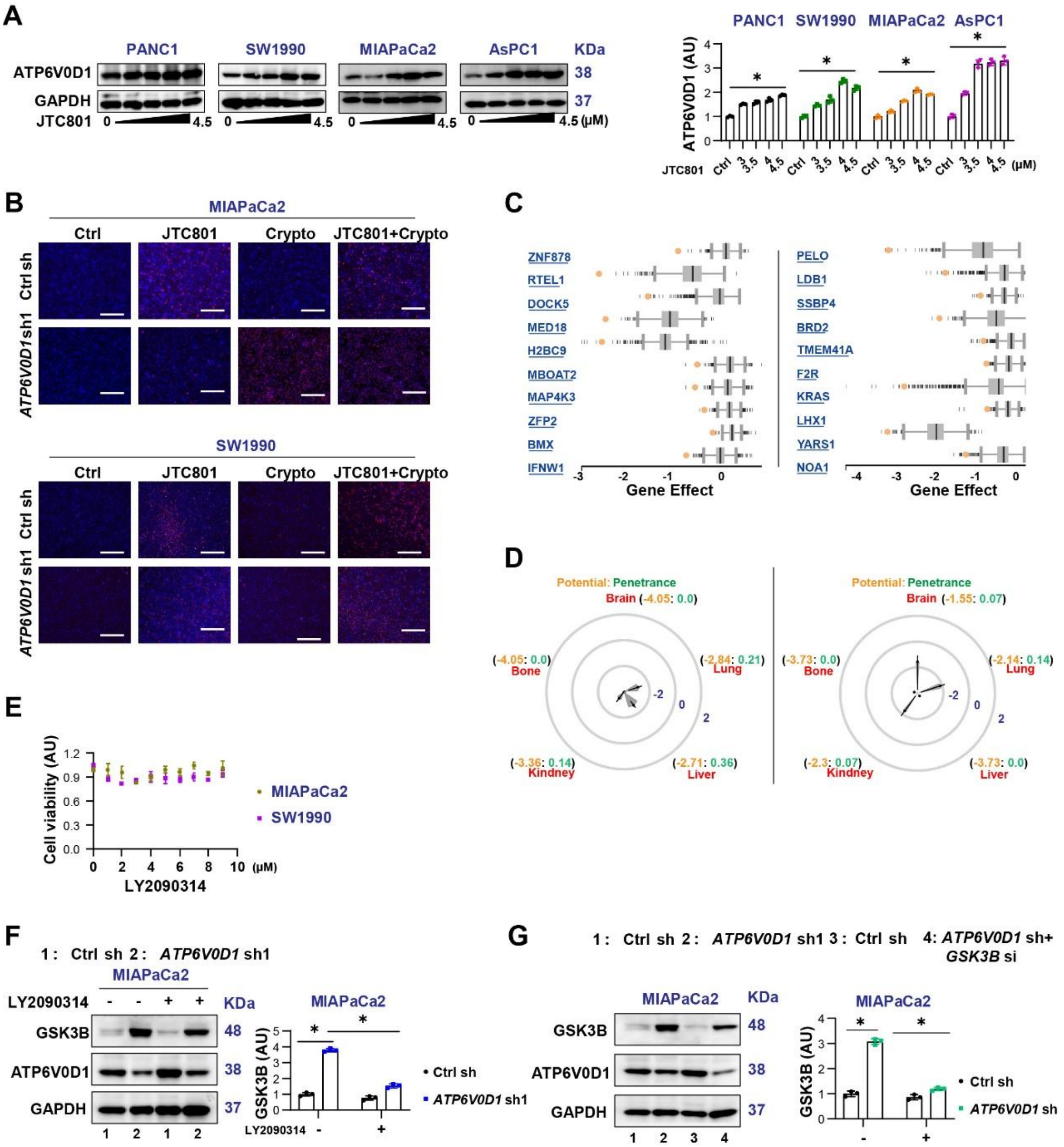

Supplementary Figure 1. Biological differences between SW1990 and MIAPaCa2 cells. (A) Western blot analysis of ATP6V0D1 protein expression under the indicated JTC801 treatments (JTC801 concentration: 3, 3.5, 4 and 4.5 μM; treatment time: 24 h). The bar chart on the right shows the semi-quantitative levels of ATP6V0D1 under JTC801 treatment. The untreated group was set to a relative value of 1; (B) Representative image corresponding to Figure 1B. Scale bar = 200 μm; (C) DepMap database analysis of the top 10 genes essential for the survival of different PDAC cell lines after genome-wide CRISPR knockout; (D) Analysis of the metastatic potential of SW1990 and MIAPaCa2 cells to different organs. Data are presented on a $\log_{10}$ scale (range: -4 to 4). ≤ -4: non-metastatic; -4 to -2: weakly metastatic, low confidence; ≥ -2: metastatic, higher confidence; (E) CCK-8 assay to assess the

cytotoxicity of LY2090314 at concentrations of 0-9 μM; (F and G) Western blot analysis of protein expression in the indicated groups (LY2090314, 5 μM; treatment time 24 h). The bar chart shows the semi-quantitative levels of the detected proteins. (A, F and G) Western blot data are representative of three independent experiments. Data are presented as mean ± SD. Statistical significance was determined using one-way ANOVA with Tukey's multiple comparisons test. Source data are provided. $^{*}P < 0.05$ was considered statistically significant; ns: not significant.

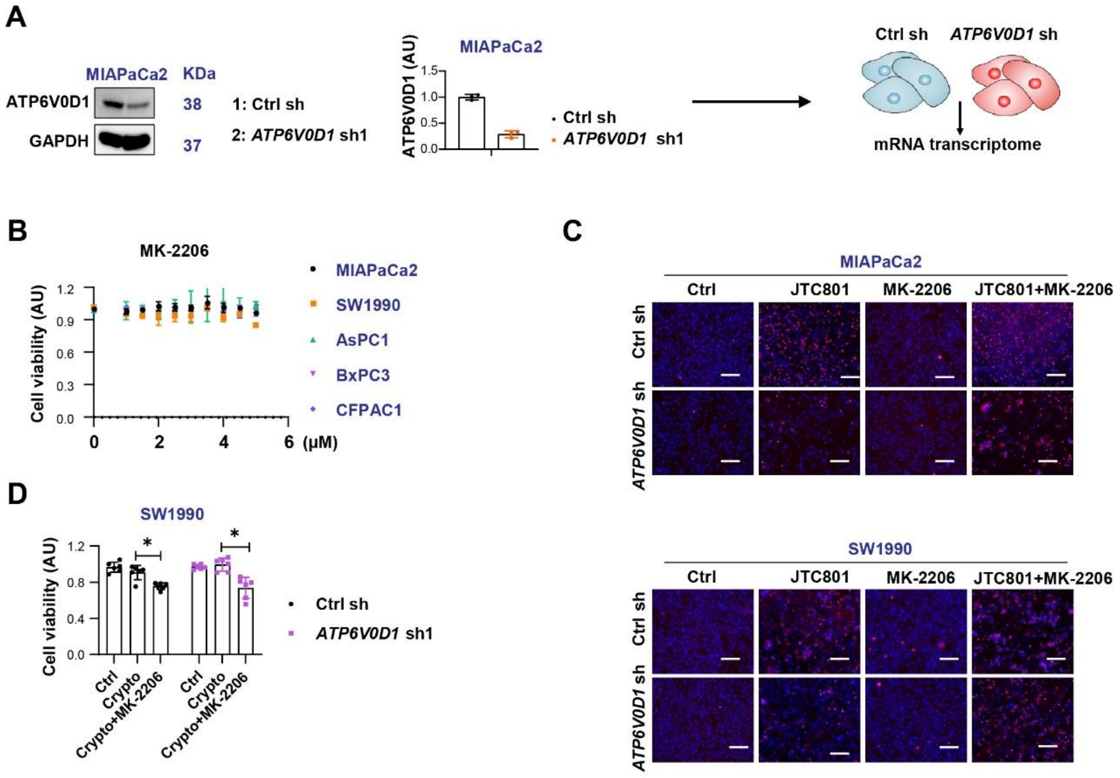

Supplementary Figure 2. AKT inhibition restores sensitivity to alkaliptosis in *ATP6V0D1*-deficient PDAC cells. (A) Western blot analysis of protein expression in the indicated groups and corresponding mRNA transcriptomic profiles. The bar chart on the right shows semi-quantitative levels of ATP6V0D1. The untreated group was set to a relative value of 1; (B) CCK-8 assay assessing the cytotoxicity of MK-2206 at various concentrations (μM); (C) Representative image corresponding to Figure 2D. Scale bar = 200 μm; (D) CCK-8 assay assessing cell viability following treatment with Crypto alone or Crypto combined with MK-2206 (Drug concentration: Crypto, 5 μM; MK-2206, 2 μM; treatment time: 24 h). (D) Data represent six biologically independent samples and are presented as mean ± SD. Statistical significance was determined using two-way ANOVA with Tukey's multiple comparisons test. (A) Western blot data are representative of three independent experiments. Data are presented as mean ± SD, and statistical significance was determined using a two-sided unpaired *t*-test. Source data are provided. $^{*}P < 0.05$ was considered statistically significant; ns: not significant.

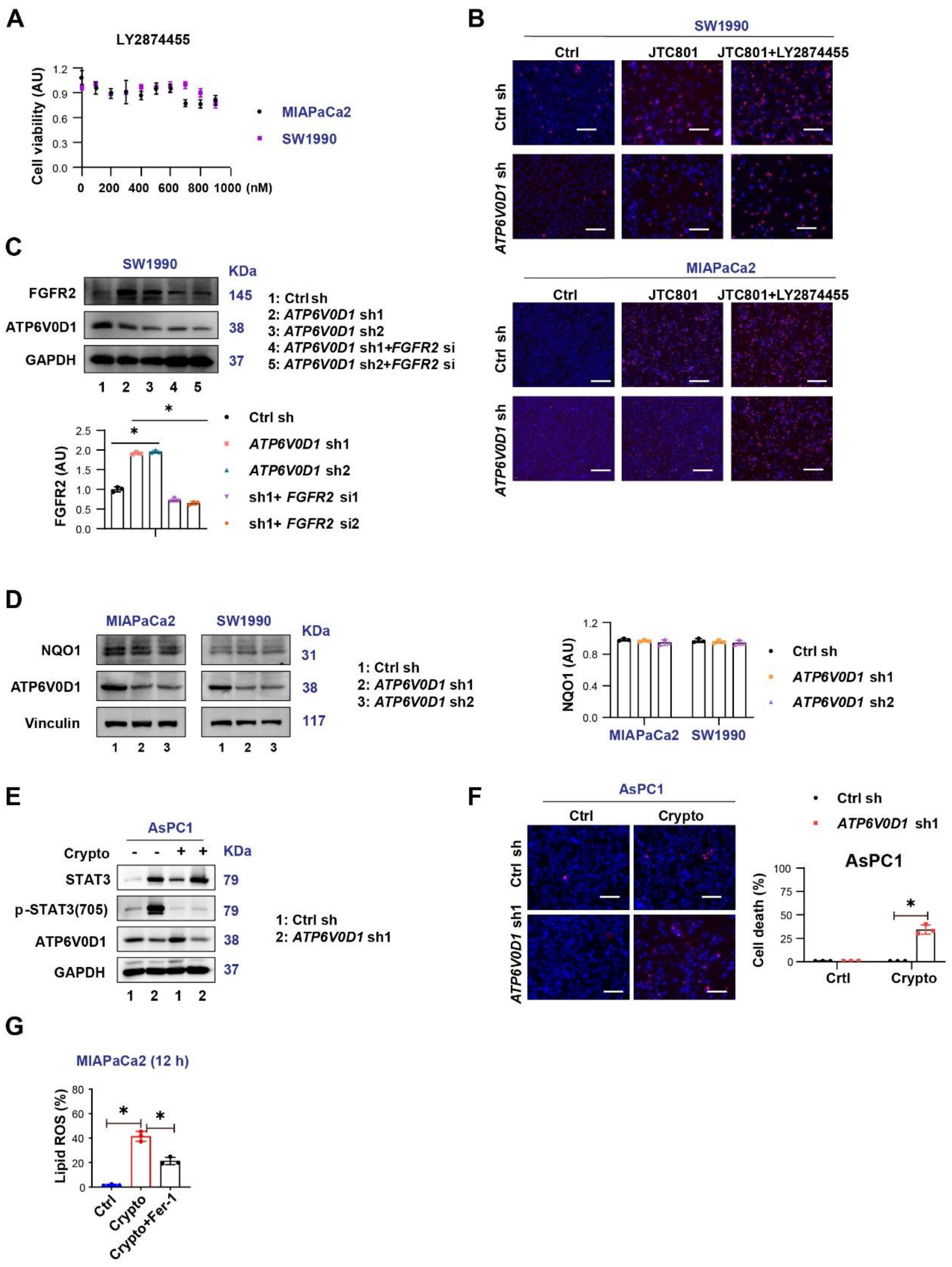

Supplementary Figure 3. FGFR2 inhibition restores sensitivity to alkaliptosis in *ATP6V0D1*-deficient PDAC cells. (A) CCK-8 assay assessing the cytotoxicity of LY2874455 at different concentrations (nM); (B) Representative image corresponding to Figure 3D. Scale bar = 200 µm; (C and D) Western blot analysis of protein expression in the indicated groups. Bar charts show semi-quantitative levels of the detected proteins. Grouping: (1) control; (2-3) *ATP6V0D1* shRNA; (4-5) *ATP6V0D1* + *FGFR2* knockdown; (E) Western blot analysis of protein expression in the indicated

groups (Drug concentration: Crypto, 5 μM; treatment time: 24 h); (F) Cell death assay assessing the proportion of dead cells following Crypto treatment (Drug concentration: 5 μM; treatment time: 24 h); (G) Lipid ROS levels in the indicated groups measured using the BODIPY 581/591 C11 probe (Drug concentration: Crypto, 5 μM; treatment time: 12 h). (C and D) Western blot data are representative of three independent experiments. Data are presented as mean ± SD. Statistical significance was determined using one-way ANOVA with Tukey's multiple comparisons test (C) or two-way ANOVA with Tukey's test (D and F). Source data are provided. $^{*}P < 0.05$ was considered statistically significant; ns: not significant.
